# Crude oligosaccharides extracted from bamboo shoots (*Dendrocalamus asper*) exhibit prebiotic activity and modulate intestinal morphology in broiler chickens without affecting growth performance

**DOI:** 10.14202/vetworld.2025.1789-1798

**Published:** 2025-06-27

**Authors:** Janjira Sittiya, Supawadee Chimtong, Narin Thongwittaya, Koh-en Yamauchi

**Affiliations:** 1 Program in Animal Science, Faculty of Animal Sciences and Agricultural Technology, Silpakorn University, Phetchaburi, Thailand; 2 Faculty of Animal Sciences and Technology, Maejo University, Sansai, Chiang Mai, Thailand; 3 Laboratory of Animal Science, Faculty of Agriculture, Kagawa University, Miki-cho, Kagawa, Japan

**Keywords:** bamboo shoots, broiler chickens, intestinal morphology, oligosaccharides, prebiotics

## Abstract

**Background and Aim:**

Bamboo shoots (BOSs) (*Dendrocalamus asper*) are rich in lignocellulosic material that can be hydrolyzed into oligosaccharides with potential prebiotic properties. This study aimed to evaluate the prebiotic and antibacterial activities of crude oligosaccharides derived from BOSs and assess their effects on growth performance, carcass traits, cecal microbiota, nutrient digestibility, and intestinal morphology in broiler chickens.

**Materials and Methods:**

In Experiment 1, BOSs were extracted using acid hydrolysis followed by enzymatic treatment, and their composition was confirmed through thin-layer chromatography and high-performance liquid chromatography. The prebiotic and antibacterial potential was evaluated *in vitro* against *Lactobacillus plantarum* and *Escherichia coli*. In Experiment 2, 240 broiler chicks (ROSS 308) were randomly allocated to four dietary groups (0%, 0.05%, 0.1%, and 0.2% BOSs) with six replicates each and reared for 42 days. Growth parameters, carcass traits, cecal bacterial counts, nutrient digestibility, and intestinal histomorphology were assessed.

**Results:**

BOSs significantly enhanced the growth of *L. plantarum* and inhibited *E. coli* (p < 0.05), demonstrating both prebiotic and antibacterial activity. However, dietary inclusion of BOSs did not significantly affect feed intake, body weight gain, feed conversion ratio, or mortality. Carcass traits, total aerobic bacteria, lactobacilli count in cecal digesta, and nutrient digestibility were not significantly altered. Notably, ileal villus height and crypt depth were significantly increased in the 0.1% BOSs group compared to the control (p < 0.05), indicating improved intestinal morphology.

**Conclusion:**

BOSs exhibited promising prebiotic activity by promoting beneficial bacteria and suppressing pathogenic bacteria. While no adverse effects on growth or nutrient utilization were observed, BOSs improved ileal histomorphology, suggesting potential benefits for gut health. Further studies are recommended to optimize dosage and purity levels to maximize functional outcomes in poultry nutrition.

## INTRODUCTION

The growing interest in probiotics and prebiotics, which support beneficial gut microbiota, has highlighted their potential as alternatives to antibiotic growth promoters in poultry nutrition. These bioactive compounds selectively stimulate favorable bacterial populations in the gastrointestinal tract, thereby enhancing gut health and overall performance in poultry [1–3]. Among these, oligosaccharides – available in both natural and synthetic forms – are widely recognized for their prebiotic properties. However, previous studies by Palaniappan *et al*. [4] and Onipe and Ramashia [5] have indicated that natural oligosaccharides may offer superior efficacy compared to commercial synthetic alternatives. Common dietary sources of natural oligosaccharides include onions, garlic, legumes, and bamboo shoots (BOSs) [6]. Sittiya and Nii [7] have identified BOSs as a particularly rich source of dietary polysaccharides and oligosaccharides, with significant prebiotic potential. Notably, BOSs stand out among plant-derived materials due to their availability from cost-effective, abundant, and renewable agricultural sources.

BOSs (*Dendrocalamus asper*) are composed of lignocellulosic material, which can be hydrolyzed into oligosaccharides through chemical, biological, or physical processes targeting cellulose and hemicellulose [8–10]. The crude oligosaccharides derived from BOSs in this study consisted of a mixture of monosaccharides, such as xylose, and oligosaccharides, primarily xylobiose. Xylo-oligosaccharides (XOSs) have garnered attention for their ability to improve poultry health and performance. These oligosaccharides act as fermentable substrates for beneficial bacteria, thereby promoting the production of short-chain fatty acids (SCFAs), improving intestinal morphology and nutrient absorption, and stimulating immune function [11–14]. Thus, prebiotic supplementation can beneficially alter the gut microbiota, ultimately influencing poultry growth and gut health. In addition, oligosaccharides may inhibit the proliferation of pathogenic bacteria by facilitating their adhesion to the oligosaccharide molecules, which supports their clearance from the gut environment [15]. This mechanism aligns with the findings of Elgeddawy *et al*. [16], who demonstrated that prebiotics could be effectively incorporated into poultry diets as a prophylactic measure against enteric pathogens. Furthermore, Xu *et al*. [17] reported that dietary supplementation with 8 g/kg of fructo-oligosaccharides (FOSs) improved broiler performance and significantly increased cecal populations of lactobacilli and bifidobacteria.

Despite the increasing interest in natural prebiotics as sustainable alternatives to antibiotic growth promoters in poultry nutrition, the exploration of crude oligosaccharides derived from BOSs remains limited. While several studies have investigated the prebiotic effects of plant-based oligosaccharides such as FOSs, mannan-oligosaccharides, and XOSs, there is a paucity of literature specifically addressing the functional efficacy of oligosaccharides extracted from *D. asper*. Moreover, existing studies have primarily focused on purified oligosaccharide fractions, whereas the biological potential of crude oligosaccharide extracts, which are more practical for feed applications, has not been thoroughly examined. In particular, the effects of BOSs on broiler performance, gut microbial populations, nutrient digestibility, and intestinal morphology under non-challenging, commercial rearing conditions have not been comprehensively evaluated. This knowledge gap limits the understanding of the practical applicability of bamboo-derived oligosaccharides in poultry feeding programs and their comparative performance relative to other prebiotic sources.

The present study was designed to evaluate the prebiotic potential and antibacterial activity of crude oligosaccharides extracted from BOSs *(D. aspe*r). The study comprised two main objectives: (1) To assess the ability of BOSs to selectively promote the growth of beneficial bacteria *(Lactobacillus plantaru*m) and inhibit pathogenic bacteria *(Escherichia col*i) *in vitr*o; and (2) to investigate the effects of dietary supplementation with varying concentrations of BOSs (0%, 0.05%, 0.1%, and 0.2%) on growth performance, carcass traits, cecal microbial populations, nutrient digestibility, and intestinal histomorphology in broiler chickens over a 42-day feeding trial. By addressing this dual objective, the study aims to provide foundational evidence for the use of BOSs as a functional feed additive that supports poultry gut health without compromising performance parameters.

## MATERIALS AND METHODS

### Ethical approval

All procedures were approved by the Institutional Animal Care and Use Committee (IACUC) of Silpakorn University, Thailand (Approval ID: 18/2564), in compliance with the ARRIVE guidelines.

### Study period and location

This study was conducted from June 2023 to November 2023 at the Faculty of Animal Sciences and Agricultural Technology Farm, Silpakorn University Phetchaburi IT Campus, Thailand.

### Preparation of crude oligosaccharides from BOSs

The BOSs (*D. asper*) used in this study were cultivated in Phetchaburi Prefecture, Thailand. All samples were cut into small pieces, oven-dried at 60°C, and then ground in a hammer mill (RT-34 model, Rong Tsong Precision Technology Co., Taiwan) equipped with a 250 μm screen.

Powdered BOSs were pretreated by chemical degradation using hydrochloric acid and hydrolytic degradation through hydrothermolysis, followed by enzymatic hydrolysis. Briefly, all the samples were stirred continuously in distilled water with 1 N hydrochloric acid for 2 h at 100°C using a magnetic stirrer with a heating plate (IKAMAG C-MAG HS7, IKA-Werke GmbH & Co. KG, Staufen, Germany). After cooling, the sample pH was adjusted to 7.0 using barium hydroxide.

Then, the samples were further hydrolyzed at 40°C for 24 h using 5 g/kg of Hemicell enzyme (Behn Meyer Chemical Co., Ltd., Thailand), which contained β-mannanase and xylanase activities. The insoluble materials were removed by centrifugation at 9,390 x *g* for 5 min. Subsequently, the supernatant was precipitated with 90% acetone and then kept overnight at 4°C in a refrigerator to precipitate oligosaccharides [18]. The BOSs were obtained after the precipitate was oven-dried at 50°C. The BOSs were then stored in polyethylene pouches and sealed at 4°C until further study. Consequently, the oligosaccharide profile and prebiotic and antibacterial activities of the BOSs were determined.

### Chemical and biological analysis of BOSs

The BOSs were chromatographically separated on a high-performance liquid chromatography system equipped with an Aminex HPX-87H column, using refractive index detection at 85°C. The mobile phase consisted of 5 mM sulfuric acid, and the separation was performed at a flow rate of 0.6 mL/min. The oligosaccharide composition was determined by comparing the average peak areas with those of mixtures of standard oligosaccharides (X1 through X3), and the results are expressed as grams per gram of sample (Table 1). Additionally, the physical properties, solubility, and pH stability of the samples were investigated (Table 1).

**Table 1:** Common properties of crude oligosaccharides derived from BOSs.

Property	Value
X1 (xylose)	41.2 g/100 g
X2 (xylobiose)	30.5 g/100 g
X3 (xylotriose)	Nd
Physical status	Crystalline solid and white color
Solubility	97% w/w at 25°C
pH stability	5.97

BOSs=Bamboo shoots

### Oligosaccharide observation and prebiotic and antibacterial activity analysis

Thin-layer chromatography (TLC) was employed to detect the presence of BOSs. The BOSs were subjected to TLC on aluminum sheet silica gel 60, F254 (Merck, Darmstadt, Germany), using a 7:2:1 (v/v/v) N-propanol/water/ammonium hydroxide mobile phase system. The spots on the chromatograms were visualized by spraying a mixture of 10:90 sulfuric acid/ethanol (v/v) [19]. The prebiotic and antibacterial activities of oligosaccharides were evaluated on two bacterial strains: *L. plantarum* and *E. coli*. Bacterial growth was monitored at an absorbance of 600 nm. Briefly, overnight culture broths from stock cultures were diluted to 0.5 optical density with de Man, Rogosa, and Sharpe (MRS) medium for *L. plantarum* and nutrient broth for *E. coli*, and then incubated in basal medium supplemented with hydrolyzed oligosaccharides. After incubation at 37°C, the relative cell growth was measured spectrophotometrically at 600 nm and compared to that in media supplemented with glucose [20, 21].

### Animals, diet, and experimental design

A total of 240 one-day-old, unsexed broiler chicks (Ross 308) were obtained from a commercial hatchery. A completely randomized design was employed, consisting of four dietary treatments with six replicate pens per treatment (10 birds/replicate pen). Birds were randomly assigned to treatment groups using a computer-generated randomization schedule to ensure baseline uniformity in body weight. The sample size (n = 60/treatment group) was calculated based on a power analysis (power = 0.8, α = 0.05) to detect a 5% difference in BWG, using previously published variance estimates for broiler performance traits. All birds were housed for 42 days in environmentally controlled pens (average temperature: 28°C–30°C, relative humidity: 65%–70%) with continuous lighting and standard biosecurity measures in place. Water was provided ad libitum. The experimental diets used for the starter period (0–21 d) and grower period (22–42 d) were in mash form and formulated according to Aviagen [22] (Table 2). The four dietary treatments were as follows: 0% (control), 0.05% BOSs, 0.1% BOSs, and 0.2% BOSs. Inclusion levels of BOSs were selected based on preliminary *in vitro* prebiotic activity assays and prior literature indicating effective oligosaccharide ranges in poultry [7].

**Table 2:** Feed compositions and calculated nutrient values of experimental diets.

Item	Starter period (0–21 days of age)	Grower period (22–42 days of age)
Ingredient (%)		
Corn	51.72	62.17
Soybean meal	38.41	28.38
Soybean oil	5.39	5.48
Monocalcium phosphate	2.11	1.74
Limestone	0.97	0.85
Premi×^1/^	0.20	0.20
Salt	0.24	0.24
D, L-methionine	0.27	0.24
Choline chloride	0.07	0.08
L-lysine	0.12	0.15
L-threonine	0.04	0.03
Sodium bicarbonate	0.20	0.20
Calculated contents		
Crude protein (%)	21.90	18.20
Metabolizable energy (kcal/kg)	3,081	3,215
Crude fiber (%)	3.41	3.04
Crude fat (%)	7.99	8.27
Calcium (%)	0.89	0.75
Available phosphorus (%)	0.45	0.38
Available lysine (%)	1.18	0.97
Available methionine (%)	0.48	0.41

^1/^Premix included the following (per kg of diet): Retinol=2.48 mg, Cholecalciferol=0.07 mg, Tocopherol=20.11 mg, Menadione=1.1 mg, Thiamine=1.4 mg, Riboflavin = 5.5 mg, Pyridoxine=1.1 mg, Cyanocobalamin = 12 μg, Niacin = 41.3 mg, Pantothenic acid=11 mg, Biotin = 41 μg, Folic acid=1.4 mg, Manganese=125 mg, iron = 282 mg, Copper=27.5 mg, Zinc=275 mg, iodine=844 μg, Selenium=250 μg

### Growth performance, carcass traits, and cecal microbial populations

The feed intake (FI), body weight gain (BWG), and mortality of the chicks in each replicate cage were recorded after 21 and 42 d. Whenever a bird was found dead, the feed and the dead bird were weighed immediately. The FI and BWG of the dead bird can be calculated and deducted from the final FI and BWG of its cage, respectively. The feed conversion ratio (FCR) and mortality rate were also calculated.

At the end of the feeding experiment, six birds with a certain average body weight (average body weight ± 100 g) from each group were used to determine the carcass traits. The wings, breasts, thighs, and drumsticks were removed and weighed individually. Their weight was expressed relative to a 100 g body weight.

Additionally, the ceca from these birds were collected for bacterial population analysis. The cecal contents from each bird were removed and placed in sterilized tubes for serial dilution. Microbial populations were determined by serial dilution (10^-2^ to 10^-8^) of cecal samples in diluent onto Petri dishes containing sterile agar. Total aerobic bacteria were counted on nutrient agar, and *Lactobacillus* was counted on MRS agar. The plates for lactobacilli were incubated anaerobically at 37°C for 48 h. The total number of aerobic cells was determined after 24 h of aerobic culture at 37 °C. The results are reported as log_10_ colony-forming units per gram (CFU/g) cecal content. All microbial plates were prepared in duplicate, and serial dilutions were performed in sterile phosphate-buffered saline (pH = 7). The limit of detection for microbial enumeration was 10^2^ CFU/g.

### Nutrient digestibility

A digestibility study was conducted using 3% chromic oxide (Cr_2_O_3_) as an indicator, and the samples were observed during the last week of two periods (weeks 3 and 6 of the experiment). The chickens were fed experimental diets mixed with Cr_2_O_3_. Feces from each replicate were collected over a 24 h period on days 21 and 42. All feed and fecal samples were stored immediately at −20°C until analysis. All samples were finely ground and analyzed for gross energy using a Parr 6200 bomb calorimeter (Parr Instrument Company, USA). The concentration of Cr_2_O_3_ in the feed and feces was determined by the method described by Fenton and Fenton [23]. The nutrient digestibility was calculated according to the following equation:

Digestibility (%) = 100 – 100 × ([Cr_2_O_3_ Diet × Nutrient Feces]/[Cr_2_O_3_ Feces × Nutrient Diet])

Nutrient digestibility values were corrected for indicator recovery using validated Cr_2_O_3_ quantification.

### Tissue sampling for intestinal morphological observation

On Day 42, another six birds per group were used for intestinal morphological observations. Immediately following decapitation, the midpoint of each intestinal segment (duodenum, jejunum, and ileum) was removed and fixed in 10% neutral-buffered formalin. After dehydration through various concentrations of alcohol, each intestinal segment was embedded in paraffin wax. A 4 μm-thick transverse section was cut and stained with hematoxylin–eosin. Subsequently, measurements were conducted using ToupView 3.7 software (Irwin, USA). The villus height, villus area, and crypt depth were measured using a light microscope as described by Iji *et al*. [24]. Eight calculations from eight sections, including villus height, villus area, crypt depth, and the villus height/crypt depth ratio, were averaged for each bird.

### Experimental structure

#### Experiment 1

The objective of this study was to estimate the number of crude oligosaccharides present in BOSs. The prebiotic and antibacterial activities of oligosaccharides were evaluated using two bacterial strains: *L. plantarum* and *E. coli*.

#### Experiment 2

Following prebiotic activity in Experiment 1, broiler diets were supplemented with BOSs to evaluate growth performance, carcass traits, cecal microbial populations, nutrient digestibility, and intestinal morphology.

##### Statistical analysis

Independent sample t-tests were used to compare the growth of bacteria between the two groups (Experiment 1). The growth performance, carcass traits, cecal microbial populations, nutrient digestibility, and intestinal morphology data (Experiment 2) from the experimental groups were tested for normality (Shapiro–Wilk test) and homogeneity of variances (Levene’s test). Parametric data were analyzed using a one-way analysis of variance followed by Tukey’s *post hoc* test. All analyses were conducted using the SPSSv19.0 (IBM Corp., NY, USA), with significance set at p < 0.05.

### RESULTS

#### Experiment 1

**Figure 1:**
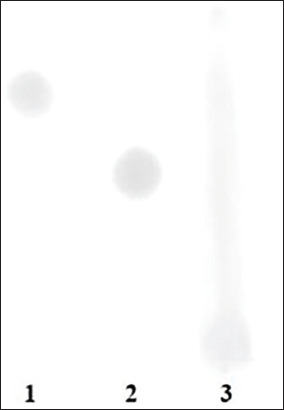
Crude oligosaccharides derived from bamboo shoots were estimated by thin-layer chromatography. Lane 1=Glucose, Lane 2=Cellobiose, Lane 3=Bamboo shoots.

As illustrated in Figure 1, the TLC analysis revealed sample spots corresponding to both monosaccharides and hexadecasaccharides, in comparison with glucose and cellobiose standards. These findings confirmed the presence of oligosaccharides in the BOSs extract.

**Figure 2:**
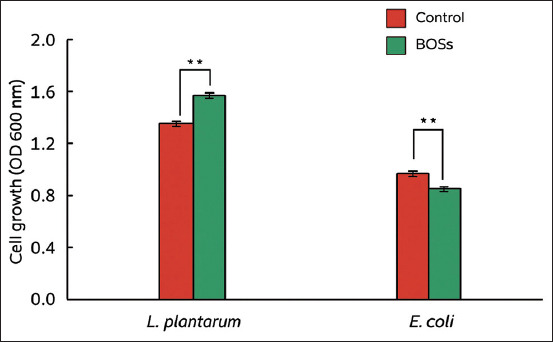
The growth rate of Lactobacillus plantarum (L. plantarum) and Escherichia coli (E. coli) of crude oligosaccharides derived from bamboo shoots (BOSs group) versus glucose medium (Control group). Values are the mean ± standard error of the mean. Asterisks (**) indicate significant differences between the Control and BOSs groups (p < 0.01).

The prebiotic and antibacterial activities of BOSs-derived oligosaccharides (BOSs) were further evaluated by monitoring the growth rates of *L. plantarum* and *E. coli* (Figure 2). Compared with the control group (glucose medium), BOSs significantly enhanced the growth of *L. plantarum*. Conversely, BOSs markedly inhibited the growth of *E. coli* relative to the control. These results suggest that the crude oligosaccharides extracted from BOSs selectively promoted beneficial bacterial growth while suppressing the growth of *E. coli*.

#### Experiment 2

The influence of dietary BOSs on growth performance and carcass traits in broiler chickens is summarized in Tables 3 and 4. No significant differences were observed in FI, BWG, FCR, or mortality rates across any treatment group during the starter, grower, or overall rearing periods (Table 3).

**Table 3:** Growth performance in broiler chickens fed diets supplemented with crude oligosaccharides derived from BOSs (n = 6).

Items	Dietary group				SEM^1/^	p-value

	Control	0.05% of BOSs	0.1% BOSs	0.2% BOSs		
Starter period (1–21 d)						
Feed intake (g)	1115.34	1099.40	1122.59	1086.00	9.44	0.535
Body weight gain (g)	943.87	946.66	955.19	927.19	6.71	0.526
Feed conversion ratio	1.18	1.16	1.17	1.17	0.01	0.820
Mortality (%)	0.00	0.00	1.67	0.00	0.43	0.443
Grower period (22–42 d)						
Feed intake (g)	2645.47	2698.76	2701.34	2662.38	32.91	0.925
Body weight gain (g)	1865.13	1872.53	1868.86	1836.72	20.82	0.936
Feed conversion ratio	1.41	1.44	1.44	1.45	0.01	0.817
Mortality (%)	0.00	2.00	1.67	0.00	0.60	0.537
Overall period (1–42 d)						
Feed intake (g)	3760.81	3798.16	3823.94	3748.38	36.02	0.886
Body weight gain (g)	2809.01	2819.19	2824.05	2763.91	25.57	0.849
Feed conversion ratio	1.33	1.34	1.35	1.35	0.01	0.863
Mortality (%)	0.00	2.00	3.33	0.00	0.95	0.548

^1/^SEM=Standard error of the mean. BOSs=Bamboo shoots

**Table 4:** Carcass traits in 42-day-old broiler chickens fed diets supplemented with crude oligosaccharides derived from BOSs (n = 6).

Items	Dietary group				SEM^1/^	p-value

	Control	0.05%BOSs	0.1% BOSs	0.2% BOSs		
Carcass (g 100 g/BW)	81.73	80.22	81.34	80.28	1.19	0.060
Thigh + drumstick motion (g 100 g/BW)	23.30	23.92	22.79	23.55	0.94	0.211
Breast (g 100 g/BW^1^)	24.06	26.84	27.54	26.75	0.55	0.118
Wing (g 100 g/BW)	8.81	8.89	9.05	8.75	0.06	0.411

^1/^SEM=Standard error of the mean. BOSs=Bamboo shoots

In addition, the weights of carcass, breast, wings, thighs, and drumsticks were not significantly affected by dietary supplementation with BOSs (p > 0.05) (Table 4).

Likewise, total aerobic bacterial counts and *Lactobacillus* populations in the cecal digesta did not differ significantly among the groups (p > 0.05) (Table 5).

**Table 5:** Viable counts of microbial in cecal digesta (log^10^ CFU/g) of broiler chickens fed diets supplemented with crude oligosaccharides derived from BOSs (n = 6).

Items	Dietary group				SEM^1/^	p-value

	Control	0.05% of BOSs	0.1% BOSs	0.2% BOSs		
Total bacterial count (aerobes)	9.83	11.29	10.14	10.90	0.34	0.448
Lactobacilli count	10.73	11.55	11.09	10.74	0.27	0.711

^1/^SEM=Standard error of the mean. BOSs=Bamboo shoots, CFU/g=Colony-forming units per gram

As presented in Table 6, no significant changes in nutrient digestibility were noted among the treatment groups during either the starter or grower phases.

**Table 6:** Nutrient digestibility in broiler chickens fed diets supplemented with crude oligosaccharides derived from BOSs (n = 6).

Items	Dietary group				SEM^1/^	p-value

	Control	0.05% of BOSs	0.1% BOSs	0.2% BOSs		
Nutrient digestibility (d 21)						
Dry matter (%)	60.17	57.93	58.29	52.21	1.52	0.311
Crude protein (%)	58.44	48.51	53.15	49.06	1.85	0.204
Gross energy (%)	68.14	65.61	66.67	61.87	1.21	0.338
Nutrient digestibility (d 42)						
Dry matter (%)	56.39	61.06	64.85	64.46	2.03	0.482
Crude protein (%)	45.14	48.91	47.60	44.28	3.04	0.962
Gross energy (%)	62.54	66.26	70.42	70.80	1.76	0.334

^1/^SEM=Standard error of the mean. BOSs=Bamboo shoots

Table 7 details the morphological parameters of intestinal segments, including villus height, villus area, crypt depth, and villus height-to-crypt depth ratio. Notably, the 0.1% BOSs group exhibited significantly increased ileal villus height and crypt depth compared with the control group (p < 0.05). Furthermore, the villus height-to-crypt depth ratio in the ileum showed a tendency to be higher in both the 0.05% and 0.1% BOSs groups than in the control (p = 0.059).

**Table 7:** Villus height, villus area, and crypt depth of the duodenum, jejunum, and ileum in 42-day-old broiler chickens supplemented with crude oligosaccharides derived from BOSs (n = 6).

Items	Dietary group				SEM^1/^	p-value

	Control	0.05% of BOSs	0.1% BOSs	0.2% BOSs		
Villus height (mm)						
Duodenum	1.51	1.60	1.72	1.66	0.050	0.560
Jejunum	1.05	0.96	1.21	1.04	0.050	0.371
Ileum	0.53^a^	0.61^ab^	0.78^b^	0.68^ab^	0.030	0.027
Villus area (mm^2^)						
Duodenum	0.28	0.32	0.26	0.25	0.013	0.310
Jejunum	0.21	0.16	0.17	0.17	0.009	0.256
Ileum	0.14	0.14	0.15	0.11	0.006	0.161
Crypt depth (mm)						
Duodenum	0.16	0.15	0.15	0.14	0.006	0.836
Jejunum	0.13	0.12	0.13	0.12	0.004	0.548
Ileum	0.09^a^	0.09^a^	0.13^b^	0.12^ab^	0.005	0.005
Villus height-to-crypt depth ratio						
Duodenum	9.94	11.57	12.53	12.23	0.552	0.371
Jejunum	8.02	8.19	9.58	9.06	0.439	0.574
Ileum	5.83	6.86	6.41	5.70	0.175	0.059

^1/^SEM=Standard error of the mean. ^a,b^Means within a row with different letters differ significantly at p *<* 0.05. BOSs=Bamboo shoots

## DISCUSSION

### Interest in natural prebiotics and the role of BOSs

Natural prebiotics from plant materials have attracted increasing interest due to consumer concerns about the safety of synthetic substances in food. To the best of our knowledge, little is known about the use of BOSs-derived oligosaccharides in broiler chickens. This study demonstrated the prebiotic activity of BOSs and the effects of dietary BOSs on growth performance, carcass traits, cecal microbial populations, nutrient digestibility, and intestinal morphology in broiler chickens. The prebiotic and antibacterial activities of BOSs in this study resulted in the promotion of beneficial bacteria and the inhibition of *E. coli*. However, BOSs did not affect growth performance, carcass traits, cecal microbial populations, or nutrient digestibility. Interestingly, the intestinal morphology of broilers improved with BOSs supplementation.

### Prebiotic activity and bacterial modulation

BOSs contain lignocellulose, a complex structure composed of cellulose and hemicellulose, which can be broken down into oligosaccharides through various treatments, including physical, physicochemical, chemical, biological, electrical, or a combination of these treatments [8]. In this study, BOSs significantly improved the growth of *L. plantarum* compared with the control (glucose medium). This result was associated with the presence of oligosaccharides in the BOSs, possibly due to the ability of BOSs to be metabolized by bacteria, suggesting that BOSs have potential prebiotic properties. These results align with previous studies by Chimtong *et al*. [25] and Azmi *et al*. [9], which found that the extraction of oligosaccharides from spent tea leaves and BOSs significantly increased beneficial bacterial growth. Interestingly, BOSs inhibited the growth of *E. coli*. This phenomenon supports the finding that a prebiotic is defined by its ability to selectively stimulate the growth of beneficial bacteria while also suppressing the growth of pathogenic bacteria [9].

### Impact on growth performance

Several studies have evaluated the effects of oligosaccharides on the growth performance of poultry. Rezaei *et al*. [26] found no effect of palm kernel expeller oligosaccharide extract on broiler growth performance (average daily gain and average daily feed intake) throughout the experimental period. Craig *et al*. [2] fed broiler chickens XOS for 29 days and reported that the FCR improved, but the BWG was not affected. De Maesschalck *et al*. [27] fed broiler chickens 0.2 g/kg XOSs from 1 to 13 days and 0.5 g/kg XOSs from 14 to 39 days, and found that the FCR improved. In the present study, broiler chickens fed BOSs at 0, 0.05, 0.1, and 0.2% from hatch to 42 d showed no significant differences in growth performance. These discrepancies may be partly due to the differences in the structures, contents, or doses of the oligosaccharides used in the experiments. The effects of oligosaccharides on poultry growth performance are variable under research conditions, suggesting potential mechanisms that require further investigation.

### Carcass traits and nutrient digestibility

The inclusion of BOSs in the present study had no influence on carcass or nutrient digestibility. Consistent with our previous study, the digestibility of gross energy was improved in the crude oligosaccharide extract from coconut milk meal during the grower period, while no difference was observed in the carcass traits of the crude oligosaccharide extract from agricultural byproduct groups [14]. Additionally, our latest research indicates that BOSs do not impact the distribution of *Lactobacillus* in the feces of layers [7]. These results are related to the number of lactobacilli in the cecal digesta in this study. The gut microbiota, a community of resident microorganisms in the digestive tracts of animals and humans, plays a crucial role in nutrient utilization and the bioconversion of food components, ultimately influencing host health [28]. In general, the intestinal microbiota plays a crucial role in the host’s well-being, facilitating the growth of beneficial bacteria that can provide essential nutrients to the host. *Lactobacilli* are known to have beneficial effects on performance and gut health and to inhibit the growth of acid-sensitive pathogenic bacteria in broilers. Therefore, controlling the growth of the intestinal microbiota is crucial for enhancing host performance. The results of the present study indicated that bacterial populations in the cecal digesta were not affected by supplementation with 0.05%–0.2% BOSs. The effects of BOSs on bacterial populations are not entirely understood. The BOSs-derived oligosaccharides (crude oligosaccharides) used in this study were composed of a mixture of monosaccharides (including xylose) and oligosaccharides (mainly xylobiose). In another study of broilers, a diet containing 0.2% XOSs during the starter period and 0.5% XOSs during grower and finisher periods increased the number of *Lactobacilli* in the colon [27]. These discrepancies may be partly due to the fact that the concentration of purified oligosaccharides was insufficient to alter the bacterial populations. Compared with crude XOSs, purified XOSs exhibit greater potency and effectiveness in promoting the growth of beneficial gut microflora [29]. Additionally, oligosaccharides may not promote the selective growth of beneficial bacteria when the indigenous population is already high before treatment [17]. However, further studies are needed to confirm whether the effect of these oligosaccharides on cecal microbial populations is due to the concentration of the purified oligosaccharides or other factors.

### Effects on intestinal morphology

Compared to the duodenum and jejunum, the ileum, located closer to the hindgut, experiences a higher pH and slower feed passage rate. This extended exposure allows the ileal microflora to exert a greater influence on nutrient absorption and digestion [30]. In the present study, the BOSs groups showed an improvement in villus height of the ileum compared to the control group. The greater ileal villus height of broilers fed BOSs might be related to the deeper crypt of the ileum in the BOSs groups. Intestinal crypt development affects the maintenance of crypt cell turnover rates and intestinal maturation. Consequently, deeper crypts result in an increased intestinal absorption surface area [31]. These results correspond with Shang *et al*. [32], who indicated that oligosaccharide supplementation may have increased the bacterial fermentation in the ileum and further increased its absorption area and stimulated the immune cells associated at the intestinal epithelial crypts. Consistent with our results, some researchers have reported that the villus height was improved in birds fed XOSs [27] or FOSs [17]. This phenomenon is supported by the findings of Su *et al*. [33], who reported that dietary supplementation with 100 g/t XOS increased the ileal villus height and the relative abundance of *Lactobacillus* and *Bifidobacterium* spp. In addition, the high ileal villus height in the BOSs groups may be due to the improved intestinal microbial environment, resulting in increased SCFA production, such as butyrate, by *Clostridium* in the cecum [11, 17, 27]. However, further studies are needed to enhance our understanding of the impact of BOSs on the production of SCFAs. Although BOSs supplementation in the diet had no significant effect on growth performance, carcass traits, cecal microbial populations, or nutrient digestibility, incorporating BOSs into the diet caused changes in the histological morphology of the intestine. The reason why the improved intestinal morphology observed in the experimental groups did not lead to BWG remains undetermined but may be related to another function, such as immunity. Chou *et al*. [34] reported similar findings with 25-OH-D_3_, where improved small intestinal morphology and protective humoral immunity against infection did not significantly affect weight gain and feed efficiency. Consistent with our previous results, oligosaccharide extract from BOSs may suppress excessive inflammatory responses during inflammation [7]. In addition, the lack of these effects under non-stressful conditions may be explained by the report of Hooge [35], who reported that most beneficial additives have the greatest impact under disease or stress conditions.

## CONCLUSION

This study demonstrated that crude oligosaccha-rides extracted from BOSs exhibit significant prebiotic and antibacterial activities. BOSs effectively enhanced the growth of *L. plantarum* while concurrently suppressing *E. coli*, affirming their potential as natural modulators of gut microbiota. However, dietary inclusion of BOSs at 0.05%, 0.1%, and 0.2% in broiler rations did not significantly alter growth performance, feed efficiency, carcass traits, cecal bacterial counts, or nutrient digestibility across a 42-day production cycle. Notably, improvements in ileal villus height and crypt depth were observed in the 0.1% BOSs group, suggest-ing a positive influence on intestinal morphology.

From a practical standpoint, BOSs may serve as a sustainable, plant-based feed additive to support gut health and intestinal integrity in poultry production systems, particularly under conditions where maintaining intestinal structure is critical. The use of BOSs aligns with global efforts to reduce reliance on antibiotic growth promoters by promoting natural alternatives derived from renewable agro-resources.

The strengths of this study lie in its comprehensive evaluation of both *in vitro* microbial effects and *in vivo* physiological responses, as well as methodical diet formulation, ethical rigor, and histological assessment. Nonetheless, the study is limited by the use of crude rather than purified oligosaccharide fractions and the absence of stress or disease challenge models, which may be necessary to elicit full functional benefits of BOSs. In addition, the relatively short duration and lack of SCFA quantification limit the mechanistic interpretation of observed histomorphological changes.

Future research should explore the effects of purified BOSs fractions, higher inclusion levels, or synergistic combinations with probiotics under pathogenic or environmental stress conditions. Investigations into SCFA production, immune modulation, and gut permeability biomarkers will further elucidate the underlying mechanisms of action.

While BOSs supplementation did not enhance growth performance, its favorable effects on gut histology and microbial modulation suggest its utility as a functional feed component. BOSs present a promising avenue for developing next-generation prebiotics in antibiotic-free poultry production systems.

## AUTHORS’ CONTRIBUTIONS

JS: Designed and conducted the study and drafted and edited the manuscript. NT and KY: Coordinated the research and provided guidance on the study. SC: Prepared oligosaccharides from BOSs and conducted prebiotic and antibacterial activity analyses. All authors have read and approved the final manuscript.
